# A New Role for Clathrin Adaptor Proteins 1 and 3 in Lipoplex Trafficking

**DOI:** 10.1371/journal.pone.0091429

**Published:** 2014-03-11

**Authors:** Justine E. Alford, Jade Gumbs, Emma C. Anderson

**Affiliations:** School of Life Sciences, University of Warwick, Coventry, United Kingdom; Cambridge University, United Kingdom

## Abstract

Intracellular protein trafficking through secretory and endocytic pathways depends on the function of adaptor proteins that bind motifs on cargo proteins. The adaptor proteins then recruit coat proteins such as clathrin, enabling the formation of a transport vesicle. While studying the role of the clathrin adaptor proteins, AP-1, AP-2 and AP-3 in viral protein trafficking, we discovered that AP-1 and AP-3 potentially have a role in successful transfection of mammalian cells with DNA-liposome complexes (lipoplexes). We showed that AP-1, -2 and -3 are not required for lipoplexes to enter cells, but that lipoplexes and/or released DNA are unable to reach the nucleus in the absence of AP-1 or AP-3, leading to minimal exogenous gene expression. In contrast, gene expression from liposome-delivered mRNA, which does not require nuclear entry, was not impaired by the absence of AP-1 or AP-3. Despite the use of lipoplexes to mediate gene delivery being so widely used in cell biology and, more recently, gene therapy, the mechanism by which lipoplexes or DNA reach the nucleus is poorly characterised. This work sheds light on the components involved in this process, and demonstrates a novel role for AP-1 and AP-3 in trafficking lipoplexes.

## Introduction

Secretory and endocytic vesicular trafficking pathways utilise clathrin adaptor proteins in the sorting and trafficking of protein cargo between compartments such as the *trans*-Golgi network (TGN), endosomes, lysosomes, plasma membrane. Adaptor proteins bind specific peptide motifs on membrane-associated cargo proteins and recruit coat proteins such as clathrin [1]. Self-assembly of a clathrin coat at a section of membrane enables the formation of a transport vesicle containing the protein cargo. The destination of the vesicle is governed by the location and identity of the adaptor protein and its interacting partners. For example, clathrin adaptor protein 2 (AP-2) is found at the plasma membrane and functions in clathrin-mediated endocytosis; AP-1 is involved in traffic between the TGN and endosomes and AP-3 is thought to function between endosomes and lysosomes [2]. These are three of the five adaptor proteins that have now been discovered, all of which are cytosolic heterotetrameric complexes composed of two large subunits (one β subunit and one of α, γ, δ, ε or ζ), a medium subunit (µ) and a small subunit (σ) [3].

In addition to well-established roles in cellular protein trafficking, AP-1 [4], AP-2 [5] and AP-3 [6] have been reported to interact with the human immunodeficiency virus type 1 (HIV-1) structural protein Gag, although in the case of AP-3 the interaction may not be direct [7]. AP-1 and AP-3 are required for viral particle release, whereas AP-2 was found to inhibit particle release. While studying the role of the APs in HIV-1 Gag trafficking, we discovered a novel role for AP-1 and AP-3 in the retrograde trafficking of DNA-liposome complexes (lipoplexes) used for cell transfection.

Gene delivery using lipoplexes is a hugely important tool in molecular and cell biology [8], and more recently in gene therapy [9]. Plasmid DNA and cationic liposomes self-assemble into complexes capable of binding to cell membranes. Intact lipoplexes have been shown to be internalised in large endocytic vesicles [10], from which they are proposed to escape into the cytoplasm at some point along the endosomal pathway [11], [12]. Efficient transgene expression depends on nuclear entry of the plasmid DNA, in order to access the transcription machinery. The data presented below shed light on the mechanism by which lipoplexes are trafficked from endosomes to the nucleus.

## Materials and Methods

### Plasmids, mRNAs and siRNAs

Plasmids used were the HIV-1 proviral plasmid pSVC21ΔBgl (HIV-1 HxB2 strain - a gift from Prof Andrew Lever), pcDNA-RL (*Renilla* luciferase) and pcDNA-GFP. Capped and polyadenylated *Renilla* luciferase and GFP mRNAs were generated by *in vitro* transcription and polydenylation using a T7 mMESSAGE mMACHINE® kit and poly(A) tailing kit (both Life Technologies) respectively. Custom siRNAs were ordered from Life Technologies: AP-1γ GCGCCUGUACAAAGCAAUU; AP-2 µ GUGGAUGCCUUUCGGGUCA; AP-3δ CCCUGUCCUUCAUUGCCAA. Additional siRNAs targeting different regions of AP-1γ and AP-3δ were ordered from Dharmacon (J-019183-06 and J-016014-06 respectively). *Silencer* negative control #2 was also used (Life Technologies, referred to in the Results as ‘con’ for control).

### Tissue Culture and Transfections

HeLa cells were maintained in Dulbecco’s Modified Eagle’s Medium (DMEM) supplemented with 10% heat inactivated foetal calf serum. Cells were seeded into 12 well plates, with or without glass coverslips, and transfected with 10 nM siRNA using Lipofectamine® 2000 the following day. 24 hours later, the cells were transfected with 1 µg/well plasmid DNA or mRNA using Lipofectamine® 2000 (Invitrogen), TransIT®-2020 (Mirus) or METAFECTENE®-FluoR (Biontex) according to the manufacturer’s instructions. For western blotting and luciferase assays, cells were harvested 24 or 48 hours after the siRNA transfection. Equal amounts of protein were analysed for luciferase activity using the luciferase assay system (Promega) or separated on 10% SDS-polyacrylamide gels and blotted onto nitrocellulose membranes. Viral and cellular proteins were detected using the following antibodies: polyclonal antiserum to HIV-1 p24/p55 (ARP432, Dr G Reid, Programme EVA, Centre for AIDS Reagents, NIBSC); anti-γ-tubulin (Sigma); anti-AP-1γ, anti-AP-2 μ and anti-AP-3δ, all from BD Biosciences (catalogue numbers 610386, 611351 and 611328 respectively).

### Confocal Fluorescence Microscopy

For microscopy, cells were fixed 6 or 24 hours after the second transfection using PBS/10% formaldehyde for 10 minutes, and permeabilised using PBS/0.5% NP40 for 10 minutes. Cover slips were mounted onto glass slides using VectaShield anti-fade mounting medium with or without DAPI, and were viewed using a Leica SP5 confocal microscope. DAPI-stained cells expressing GFP were analysed using particle counting of single channel images in Image J. METAFECTENE®-FluoR transfected cells expressing GFP were analysed by two different people using a cell counter plug-in in Image J.

### Statistical Testing

Results from luciferase assays and confocal microscopy were analysed using the unpaired Student’s t-test. The effect of each siRNA treatment was compared to the no siRNA (−) sample. Statistically significant differences (p<0.05) are indicated by asterisks in the figures and the p values are stated in the figure legends.

## Results

### Knockdown of AP-1 or AP-3 Reduces Exogenous Gene Expression from Plasmid DNA

**Figure 1:**
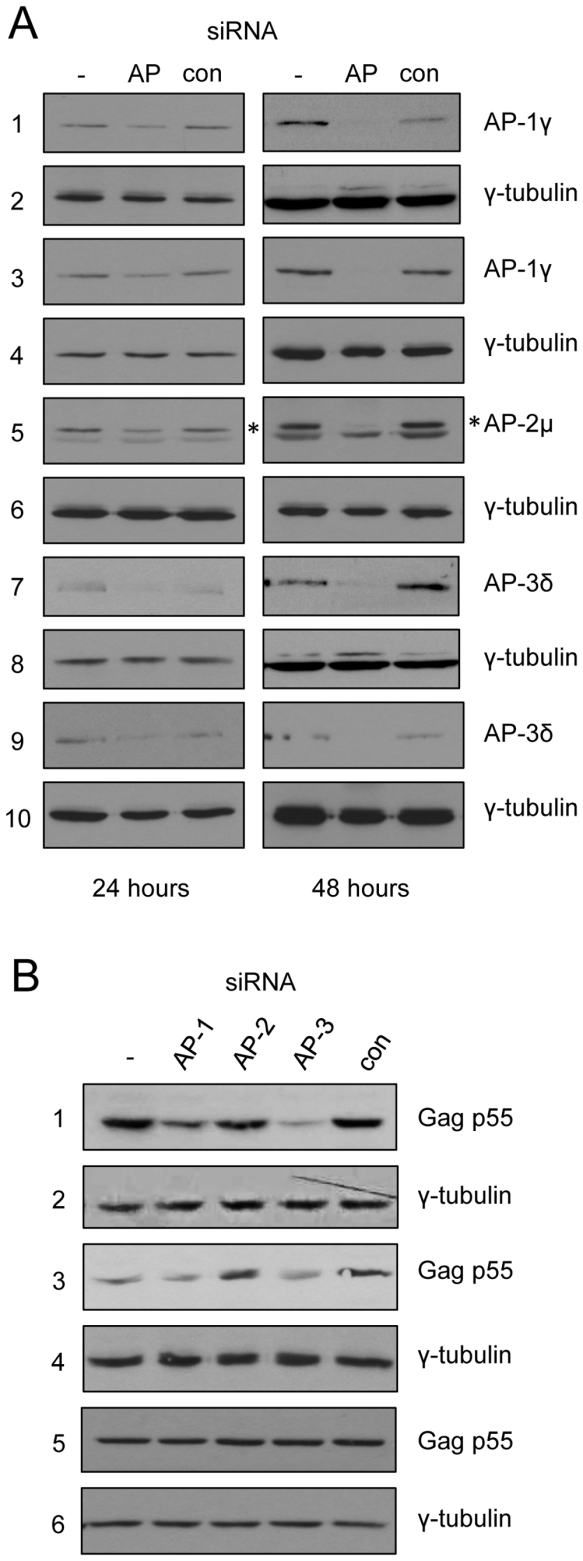
siRNA-mediated knockdown of AP-1 or AP-3 reduces HIV-1 Gag gene expression. HeLa cells were harvested 24 or 48 hours post-transfection with no siRNA (−), a control siRNA (con) or siRNAs against AP-1γ, AP-2 µ or AP-3δ. **A.** Western blot of cell lysates harvested 24 hours (left panels) or 48 hours (right panels) post-siRNA transfection, probed with antibodies against AP-1γ (panels 1 and 3, siRNAs from Life Technologies and Dharmacon respectively), AP-2 µ (panel 5, asterisk indicates the band corresponding to AP-2 µ), AP-3δ (panels 7 and 9, siRNAs from Life Technologies or Dharmacon respectively) or γ-tubulin (panels 2, 4, 6, 8, 10 which are controls for the panel above in each case). **B.** Western blot of cell lysates probed with antibodies against HIV-1 Gag p55 (panels 1, 3, 5) or γ-tubulin (panels 2, 4, 6) when cells were transfected with an HIV-1 proviral plasmid 24 hours after siRNAs (panels 1–4) or co-transfected with proviral plasmid and siRNAs for 48 hours (panels 5 and 6). Panels 3 and 4 are a repeat of the experiment shown in panels 1 and 2, but using different siRNAs to AP-1γ and AP-3δ.

In order to study trafficking of the structural protein of human immunodeficiency virus type 1 (HIV-1 Gag p55) in the absence of clathrin adaptor proteins, we utilised siRNA-mediated knockdown to reduce the cellular levels of AP-1, AP-2 and AP-3. It has previously been demonstrated that siRNAs directed against AP-1γ, AP-2 µ or AP-3δ significantly reduce the level of the respective subunits and as a consequence reduce the level of the functional heterotetrameric complex [4], [13]. A visible reduction of the AP subunits was observed 24 hours after siRNA transfection (Figure 1A, left panels) and 48 hours after siRNA transfection the cellular levels of AP-1γ, AP-2 µ and AP-3δ were substantially reduced (Figure 1A, right panels). 24 hours after transfection with these siRNAs, a negative control siRNA (con), or a mock transfection (−), HeLa cells were transfected with an HIV-1 proviral plasmid. The cells were harvested 24 hours after proviral plasmid transfection (48 hours after siRNA transfection) and analysed by western blotting. Figure 1B shows that knockdown of AP-1 or AP-3 (with either of two different siRNAs), but not AP-2, reduced the expression of HIV-1 Gag p55 (panels 1 and 3) but endogenous gamma tubulin (panels 2 and 4) was unaffected. However, if the cells were co-transfected with the siRNAs and proviral plasmid and harvested after 48 hours, knockdown of AP-1 and AP-3 did not affect the levels of Gag p55 (panel 5). This suggested that reduced AP-1 and -3 levels did not result in increased turnover of the Gag protein. Having also seen the same results when probing the cell lysates for the HIV-1 Nef protein (data not shown), we began to suspect that knockdown of AP-1 and AP-3 may affect proviral transfection efficiency rather than Gag gene expression.

**Figure 2:**
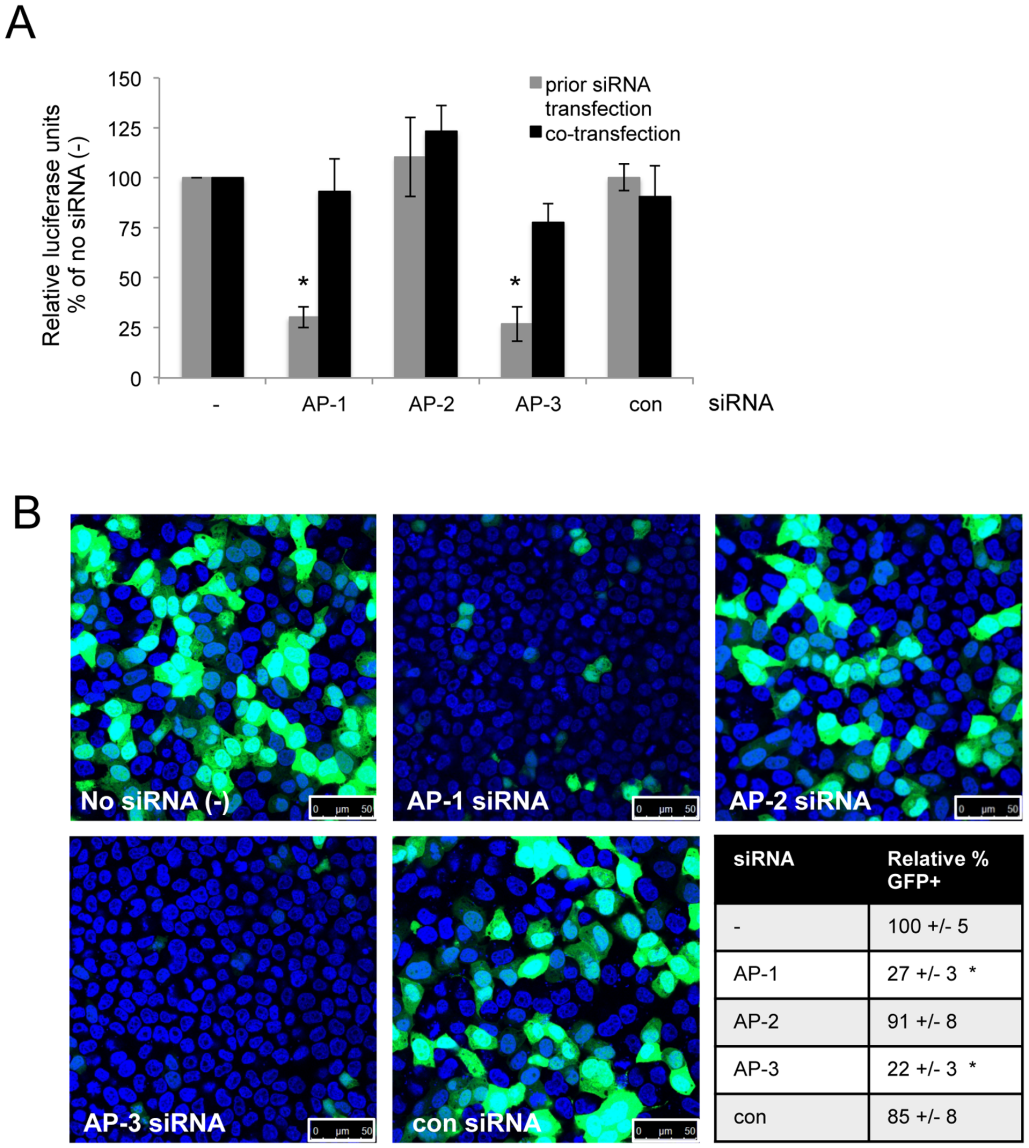
siRNA-mediated knockdown of AP-1 or AP-3 reduces exogenous gene expression. **A.** Relative luciferase activity of lysates from cells transfected with a *Renilla* luciferase plasmid 24 hours after siRNAs (grey bars) or co-transfected with plasmid and siRNAs (black bars). The data are from three or four independent experiments, error bars represent the standard error of the mean and asterisks indicate significant differences from no siRNA (−) samples (p = 9.5×10^−5^ and 5.1×10^−4^ for prior transfection of AP-1 and AP-3 siRNAs respectively). **B.** GFP fluorescence (green) of cells transfected with a GFP plasmid 24 hours after siRNAs. DAPI nuclear stain is shown in blue and 50 µm scale bars are shown in each image. The percentage of GFP positive cells was counted in ten fields of view (1000–2000 cells) for each siRNA treatment, from up to four independent experiments. The mean percentage of GFP positive cells relative to the no siRNA samples (−), +/− standard error, is shown. Asterisks indicate significant differences from no siRNA (−) samples (p = 2.7×10^−11^ and 5.4×10^−11^ for AP-1 and AP-3 siRNAs respectively).

We then went on to test whether knockdown of AP-1, -2 or -3 affected expression of reporters on other plasmids, such as *Renilla* luciferase and GFP. Figure 2A shows that expression of *Renilla* luciferase was significantly reduced by prior transfection with AP-1 and AP-3 siRNAs (grey bars) but not by co-transfection (black bars), and figure 2B shows that prior transfection with AP-1 and AP-3 siRNAs also significantly reduced the percentage of GFP-positive cells visualised by confocal fluorescence microscopy. We saw the same results with two different lipofection reagents (Lipofectamine® 2000 and TransIT®-2020).

Since AP-2 knockdown did not reduce transfection efficiency, this suggests that lipoplex cell entry is not dependent on clathrin-mediated endocytosis, a conclusion that is supported by work in other cell lines [14].

### Knockdown of AP-1 or AP-3 does not Prevent Lipoplexes Entering Cells, but Affects their Intracellular Fate

**Figure 3:**
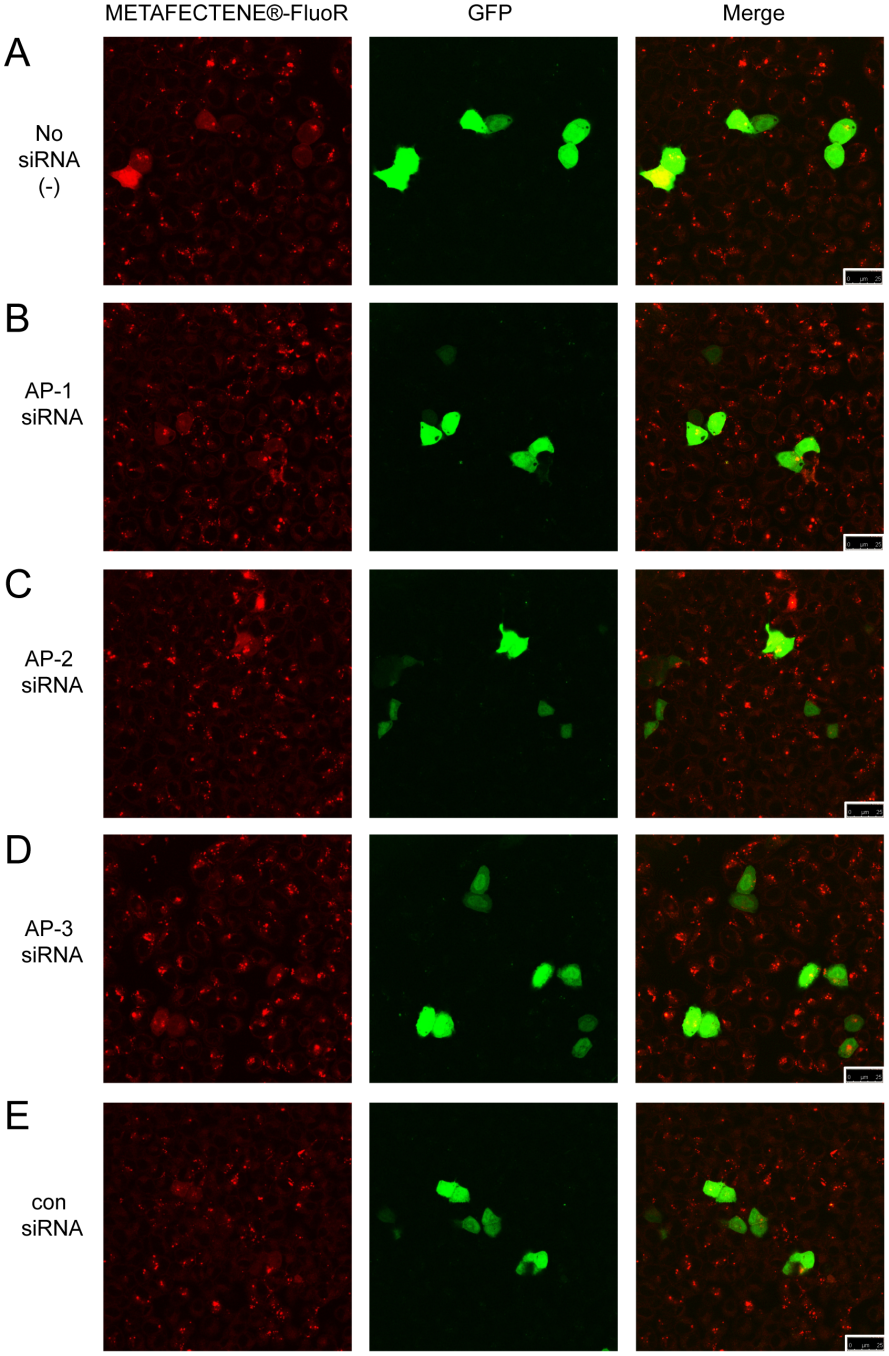
AP-1 and AP-3 are not required for cellular uptake of lipoplexes. HeLa cells were transfected with no siRNA (−, **A**), siRNAs against AP-1γ, AP-2 µ or AP-3δ (**B–D** respectively) or a control siRNA (con,**E**). 24 hours later, the cells were transfected with GFP plasmid using a rhodamine B-labelled lipofection reagent, and fixed after 6 hours. Rhodamine fluorescence from METAFECTENE®-FluoR (red) is shown in the left panels, GFP fluorescence (green) is shown in the middle panels, and a merged image of the two channels is shown in the right panels. 25 µm scale bars are highlighted in the bottom right of the merged images.

To investigate the role of the APs in the transfection process, we used METAFECTENE®-FluoR, a lipofection reagent containing a covalently-linked rhodamine B dye. HeLa cells were transfected with siRNAs against AP-1, -2, -3, a negative control siRNA (con) or no siRNA (−) 24 hours prior to transfection with lipoplexes formed from METAFECTENE®-FluoR and a GFP-encoding plasmid. A combination of confocal fluorescence microscopy to visualise the METAFECTENE®-FluoR and GFP, and bright field microscopy (not shown) to visualise the cells was used, as nuclear stains were found to mask the METAFECTENE®-FluoR signal. 6 hours after transfection, lipoplexes (red) could be seen as punctate cytoplasmic spots inside all cells (Figure 3, left panels), regardless of prior siRNA treatment, indicating that endocytic uptake of lipoplexes was not reduced by AP knockdown.

**Figure 4:**
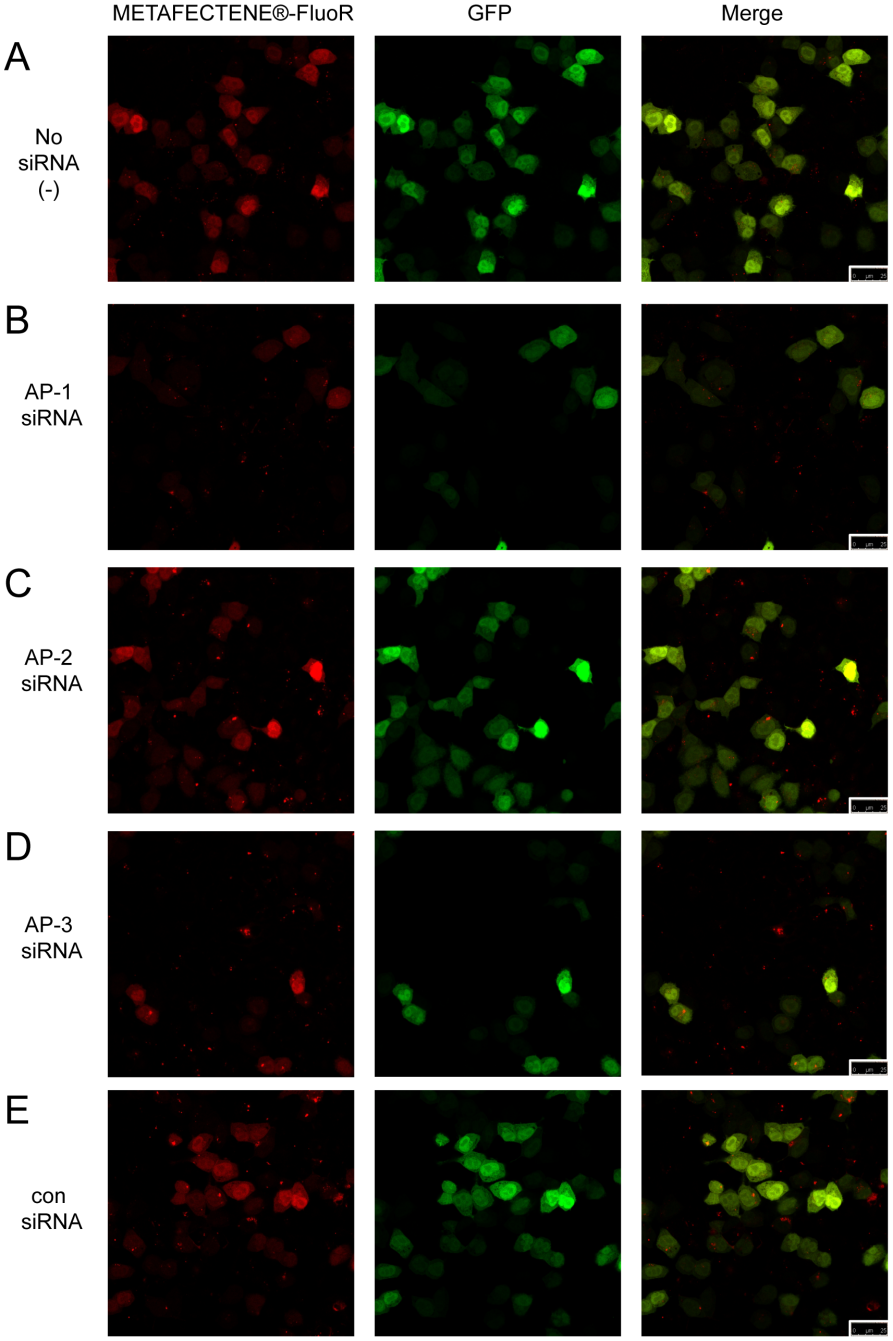
AP-1 and AP-3 are required for nuclear localisation of lipoplexes. HeLa cells were transfected with no siRNA (−, **A**), siRNAs against AP-1γ, AP-2 µ or AP-3δ (**B–D** respectively) or a control siRNA (con, **E**). 24 hours later, the cells were transfected with GFP plasmid using a rhodamine B-labelled lipofection reagent, and fixed after another 24 hours. Rhodamine fluorescence from METAFECTENE®-FluoR (red) is shown in the left panels, GFP fluorescence (green) is shown in the middle panels, and a merged image of the two channels is shown in the right panels. 25 µm scale bars are highlighted in the bottom right of the merged images.

**Figure 5:**
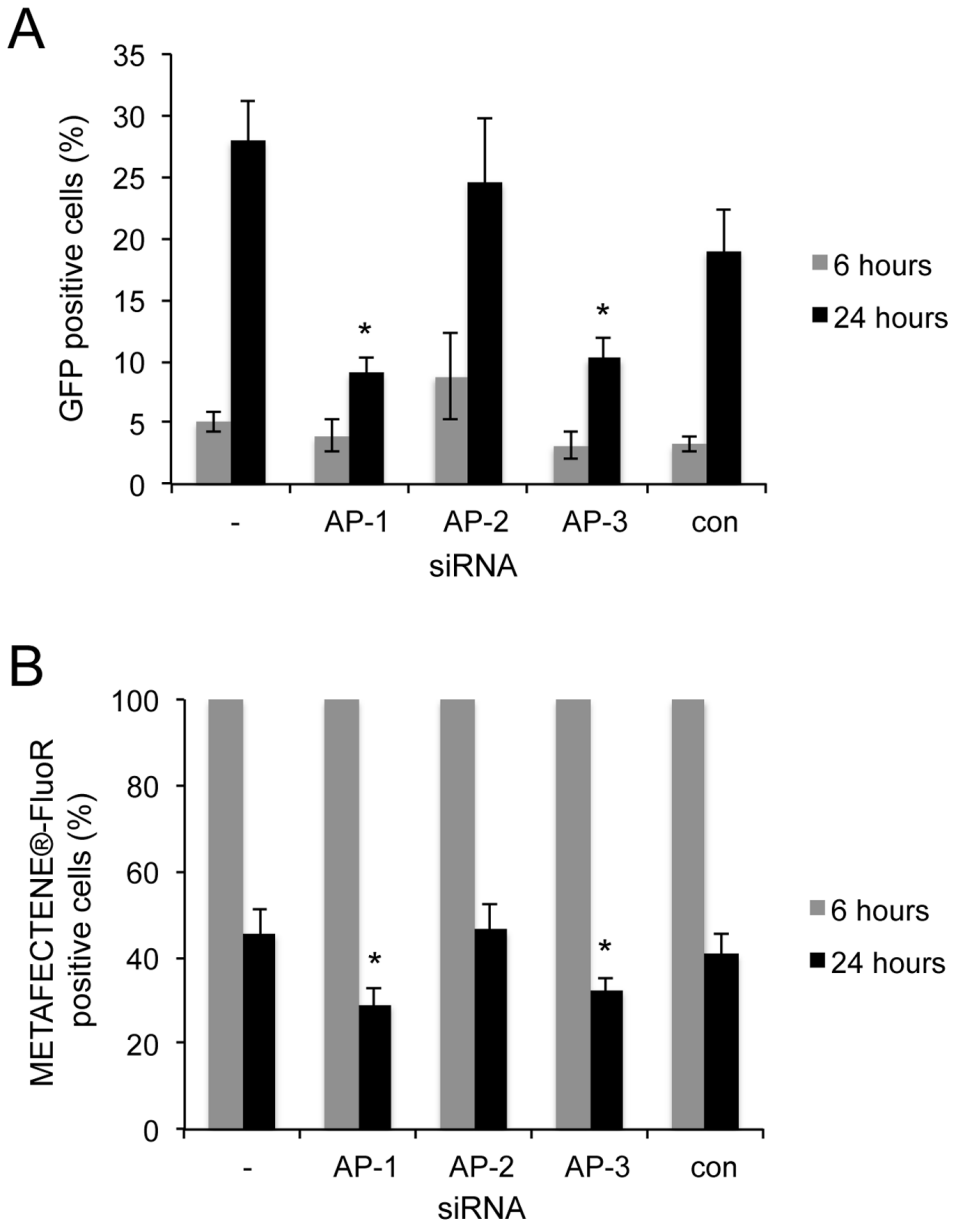
siRNA-mediated knockdown of AP-1 or AP-3 reduces GFP and METAFECTENE®-FluoR fluorescence in transfected cells. Quantitation of the experiments shown in figures 3 and 4. The percentage of GFP positive cells (**A**) and METAFECTENE®-FluoR positive cells (**B**) was counted at 6 hours (grey bars) and 24 hours (black bars) after GFP transfection. For each siRNA treatment at each time point, between five and nine fields of view (600–900 cells) from up to three independent experiments were analysed. Error bars represent the standard error of the mean. Asterisks indicate significant differences to no siRNA (−) samples (**A**, p = 5.7×10^−5^ and 3.1×10^−4^ for AP-1 and AP-3 siRNAs at 24 hours respectively; **B**, p = 0.013 and 0.026 for AP-1 and AP-3 siRNAs at 24 hours respectively).

At this early time point, a few cells were expressing GFP (Figure 3, middle panels); notably these cells showed a diffuse METAFECTENE®-FluoR signal in both cytoplasm and nucleus. This suggests that the lipoplexes had escaped endosomes and reached the nucleus, enabling expression of GFP from the introduced plasmid. Cells were also visualised 24 hours after transfection with METAFECTENE®-FluoR and GFP-encoding plasmid (Figure 4). While the numbers of GFP-positive cells were too low at 6 hours (3–8%) to observe any significant effects of prior siRNA treatment on GFP expression (Figure 5A, grey bars), at 24 hours higher numbers of cells were expressing GFP (around 20–30%) that had received no siRNA (−), AP-2 siRNA, or con siRNA (Figure 4A, C, E and Figure 5A, black bars). The GFP-positive cells all showed a diffuse METAFECTENE®-FluoR signal throughout the cell. In contrast, fewer of the cells (around 10%) that had received AP-1 or AP-3 siRNA (Figure 4B and D respectively, and Figure 5A, black bars) were GFP-positive at 24 hours. Whereas all cells were METAFECTENE®-FluoR positive at 6 hours (Figure 5B, grey bars), by 24 hours 40–45% of the cells which received no siRNA (−), AP-2 siRNA or con siRNA were positive, compared to around 30% of the cells which had received AP-1 or AP-3 siRNA (Figure 5B, black bars). The GFP-negative cells either showed punctate spots of METAFECTENE®-FluoR or no signal. The reduction of liposome staining in the absence of AP-1 or AP-3 suggests that if lipoplexes are unable to be trafficked to the nucleus, they are degraded or recycled out of the cell.

### AP-1 or AP-3 are not Required for Exogenous Expression of mRNA

**Figure 6:**
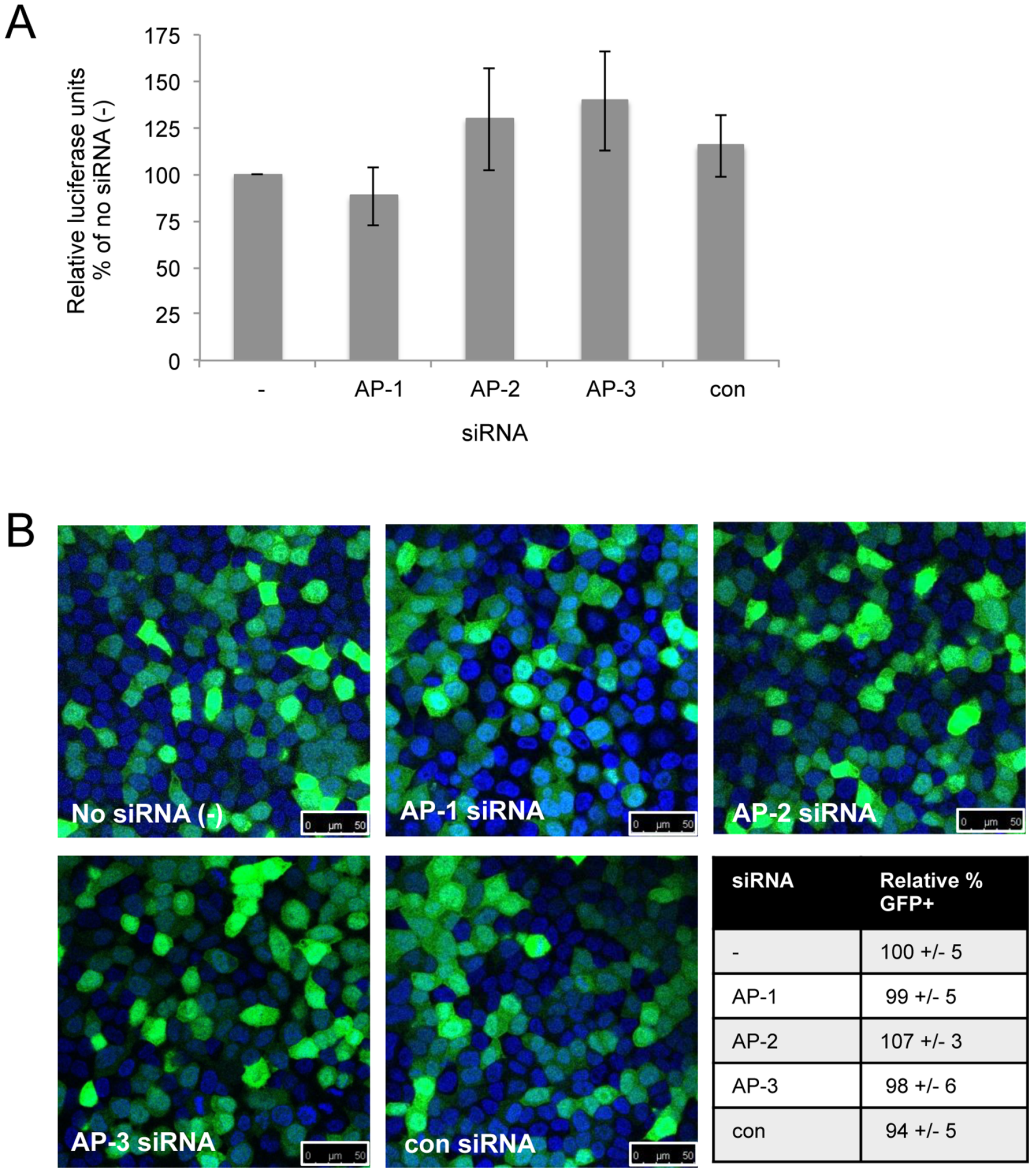
AP-1 and AP-3 are not required for transfection with mRNA. HeLa cells were transfected with no siRNA (−), siRNAs against AP-1γ, AP-2 µ or AP-3δ or a control siRNA (con). **A.** Relative luciferase activity of lysates from cells transfected with *Renilla* luciferase mRNA 24 hours after siRNAs and harvested after a further 24 hours. The data are from three independent experiments and error bars represent the standard error of the mean. **B.** GFP fluorescence (green) of cells transfected with GFP mRNA 24 hours after siRNAs and fixed after a further 24 hours. DAPI nuclear stain is shown in blue and 50 µm scale bars are shown in each image. The percentage of GFP positive cells was counted in ten fields of view (1000–2000 cells) for each siRNA treatment, from three independent experiments. The mean percentage of GFP positive cells relative to the no siRNA samples (−), +/− standard error, is shown.

The results of transfections with METAFECTENE®-FluoR suggested that AP-1 and AP-3 are required for the transport of endocytosed lipoplexes to the nucleus. We therefore tested whether knockdown of the APs would affect expression from reporter mRNAs, which do not require access to the nucleus. HeLa cells were transfected with siRNAs against AP-1, -2, -3, a negative control siRNA (con) or no siRNA (−) 24 hours prior to transfection with lipoplexes formed from Lipofectamine® 2000 and *in vitro* transcribed *Renilla* luciferase or GFP mRNA. The cells were harvested or fixed 24 hours after the second transfection and analysed for luciferase activity or GFP fluorescence. Figure 6A shows that there were no significant effects of prior siRNA treatment on expression of the *Renilla* luciferase mRNA. Fluorescence microscopy of cells transfected with GFP mRNA (Figure 6B) showed no effect of AP knockdown on the percentage of GFP-positive cells. These results suggest that AP-1 and -3 are not required for escape of lipoplexes from endosomes, since mRNAs are released and able to be translated in the cytoplasm in the absence of these proteins.

## Discussion

In studying the role of clathrin adaptor proteins in the mechanism of HIV-1 particle assembly, we have discovered that AP-1 and AP-3 are required for the efficient transfection of mammalian cells with plasmid DNA. Prior treatment of cells with siRNAs that knocked down AP-1 or AP-3, but not AP-2, significantly reduced gene expression from exogenous plasmids. Visualisation of DNA-lipoplexes with rhodamine-labelled cationic liposomes showed that the block to transfection occurred after endocytic uptake of lipoplexes. Successful transfection of the cells with mRNA-lipoplexes demonstrated that the knockdown of AP-1 or AP-3 did not prevent endosomal escape of lipoplexes or the activity of the protein synthesis machinery in the cytoplasm. The fact that endogenous gene expression was not affected by knockdown of AP-1 or AP-3 suggests that the activity of transcription and mRNA export pathways are also not impaired. Together, these results suggest that AP-1 and AP-3 are required for trafficking of lipoplexes or released DNA to the nucleus, or nuclear entry itself.

Upon transfection of dividing cells, nuclear entry of plasmid DNA most likely occurs on disintegration of the nuclear membrane during cell division. We did not observe any effect of AP-1 or AP-3 knockdown on cell division that would suggest this to be a factor in the reduced transfection efficiency. Other methods of nuclear entry such as the binding of nuclear localisation signal (NLS)-containing transcription factors to plasmid DNA have also been proposed to play a role in efficient cell transfection [15], and we can not rule out a role for AP-1 and AP-3 in the nuclear transport of transcription factors, although this might also be expected to affect endogenous gene expression.

The importance of delivery of plasmid DNA to the perinuclear region during transfection is supported by data from DNA microinjection experiments, which showed that the efficiency of transfection correlated with the proximity of the injection site to the nucleus [16]. Since cytoplasmic DNA is degraded quite rapidly (half life around 90 minutes in HeLa cells [17]), and plasmid DNA does not move freely in the cytosol [18], delivery of DNA close to the nucleus is likely to aid the number of plasmids available for nuclear entry and subsequent gene expression. In the context of lipoplex-mediated gene delivery, successful transfection may require the transport of plasmid DNA to the perinuclear region either within endocytic vesicles, or in released lipoplexes or as free DNA. Live imaging of oligonucleotide-lipoplexes in HeLa cells recently showed retrograde movement of some lipoplex-containing endosomes followed by release of the oligonucleotides they carried [18].

Adaptor protein complexes have well characterised roles in sorting protein cargo into vesicles for trafficking between cellular membrane compartments, with AP-1 functioning in TGN-endosome transport and AP-3 in endosome-lysosome transport. The heterotetrameric complexes have binding sites for membrane phosphoinositides, peptide motifs in the cargo protein, and vesicle coat proteins. A link between protein sorting and vesicle movement remained unclear until 2009, when both AP-1 and AP-3 were reported to interact with members of the kinesin superfamily to facilitate movement of vesicles along microtubules: AP-1 as part of a Gadkin/AP-1/kinesin KIF5 complex [19], and AP-3 directly with KIF3A [20]. However, both these kinesins are plus-end-directed motor proteins, facilitating anterograde trafficking. Microtubules have been shown to play a role in the trafficking of microinjected plasmids during transfection, with the minus-end-directed motor protein dynein, along with other cell proteins, proposed to mediate the interaction between plasmids and microtubules [21], [22]. Recently, studies with a very short double stranded DNA molecule identified the minus-end-directed kinesin KIFC1 as being involved in the active transport of naked DNA along microtubules [23]. Future work will investigate whether AP-1 and AP-3 interact with the minus-end directed KIFC1 to mediate the retrograde trafficking of lipoplex-containing vesicles or released lipoplexes or DNA along microtubules to the nucleus.

In conclusion, this is the first report of a novel role for AP-1 and AP-3 in the retrograde trafficking of DNA or lipoplexes during transfection. In addition to identifying components of the cellular trafficking machinery that are utilised for gene delivery, this study highlights potential pitfalls when investigating the trafficking of exogenous proteins.
